# Ionizing Radiation Changes the Electronic Properties of Melanin and Enhances the Growth of Melanized Fungi

**DOI:** 10.1371/journal.pone.0000457

**Published:** 2007-05-23

**Authors:** Ekaterina Dadachova, Ruth A. Bryan, Xianchun Huang, Tiffany Moadel, Andrew D. Schweitzer, Philip Aisen, Joshua D. Nosanchuk, Arturo Casadevall

**Affiliations:** 1 Department of Nuclear Medicine, Albert Einstein College of Medicine, Bronx, New York, United States of America; 2 Microbiology and Immunology, Albert Einstein College of Medicine, Bronx, New York, United States of America; 3 Physiology and Biophysics, Albert Einstein College of Medicine, Bronx, New York, United States of America; 4 Medicine, Albert Einstein College of Medicine, Albert Einstein College of Medicine, Bronx, New York, United States of America; Newcastle University, United Kingdom

## Abstract

**Background:**

Melanin pigments are ubiquitous in nature. Melanized microorganisms are often the dominating species in certain extreme environments, such as soils contaminated with radionuclides, suggesting that the presence of melanin is beneficial in their life cycle. We hypothesized that ionizing radiation could change the electronic properties of melanin and might enhance the growth of melanized microorganisms.

**Methodology/Principal Findings:**

Ionizing irradiation changed the electron spin resonance (ESR) signal of melanin, consistent with changes in electronic structure. Irradiated melanin manifested a 4-fold increase in its capacity to reduce NADH relative to non-irradiated melanin. HPLC analysis of melanin from fungi grown on different substrates revealed chemical complexity, dependence of melanin composition on the growth substrate and possible influence of melanin composition on its interaction with ionizing radiation. XTT/MTT assays showed increased metabolic activity of melanized *C. neoformans* cells relative to non-melanized cells, and exposure to ionizing radiation enhanced the electron-transfer properties of melanin in melanized cells. Melanized *Wangiella dermatitidis* and *Cryptococcus neoformans* cells exposed to ionizing radiation approximately 500 times higher than background grew significantly faster as indicated by higher CFUs, more dry weight biomass and 3-fold greater incorporation of ^14^C-acetate than non-irradiated melanized cells or irradiated albino mutants. In addition, radiation enhanced the growth of melanized *Cladosporium sphaerospermum* cells under limited nutrients conditions.

**Conclusions/Significance:**

Exposure of melanin to ionizing radiation, and possibly other forms of electromagnetic radiation, changes its electronic properties. Melanized fungal cells manifested increased growth relative to non-melanized cells after exposure to ionizing radiation, raising intriguing questions about a potential role for melanin in energy capture and utilization.

## Introduction

The term “melanin” originates from *melanos* - a Greek word for black. Melanin is a high molecular weight pigment, ubiquitous in nature, with a variety of biological functions [1]. Many fungi constitutively synthesize melanin [2], which is likely to confer a survival advantage in the environment [3] by protecting against UV and solar radiation [reviewed in 4]. Melanized microorganisms inhabit some remarkably extreme environments including high altitude, Arctic and Antarctic regions [5]. Most dramatically, melanized fungal species colonize the walls of the highly radioactive damaged reactor at Chernobyl [6] and surrounding soils [7]. These findings, and the laboratory observations of the resistance of melanized fungi to ionizing radiation [8], [9], suggest a role for this pigment in radioprotection.

The role of melanin in microorganisms living in high electromagnetic radiation fluxes is even more intriguing when the pigment is considered from a paleobiological perspective. Many fungal fossils appear to be melanized [10], [11]. Melanized fungal spores are common in the sediment layers of the early Cretaceous period when many species of animals and plants died out which coincides with the Earth's crossing the “magnetic zero” resulting in the loss of its : “shield” against cosmic radiation [12]. Additionally, radiation from a putative passing star called Nemesis has been suggested as a cause of extinction events [13]. The proliferation of melanotic fungi may even have contributed to the mass extinctions at the end of Cretaceous period [14]. A symbiotic association of plants and a melanotic fungus that allows for extreme thermotolerance has been attributed to heat dissipating properties of melanin [15]. Melanotic fungi inhabit the extraordinarly harsh climate of Antarctica [5]. Hence, melanins are ancient pigments that have probably been selected because they enhance the survival of melanized fungi in diverse environments and, perhaps incidentally, in various hosts. The emergence of melanin as a non-specific bioprotective material may be a result of the relative ease with which these complicated aromatic structures can be synthesized from a great variety of precursors [2], [4], [5], [16]–[23].

Despite the high prevalence of melanotic microorganisms in radioactive environments, it is unlikely that melanin is synthesized solely for the purposes of protection (shielding) from ionizing radiation. For example, in high elevation regions inhabited by melanotic fungi the background radiation levels are approximately 500–1,000 higher than at sea level, which amounts to a dose of 0.50–1.0 Gy/year. Since the overwhelming majority of fungi, melanized or not, can withstand doses up to 1.7×10^4^ Gy [9], there is no apparent requirement for melanin as a radiation protector. On the other hand, biological pigments play a major role in photosynthesis by converting the energy of light into chemical energy. Chlorophylls and carotenoids absorb light of certain wavelengths and help convert photonic energy into chemical energy during photosynthesis. Given that melanins can absorb visible and UV light of all wavelengths [16], we hypothesized that exposure to ionizing radiation would change the electronic properties of melanin and affect the growth of melanized microorganisms. Here we report the results of physico-chemical investigations of melanin electronic properties after radiation exposure and the enhanced growth of melanized fungi under conditions of radiation flux.

## Results

### Chemical composition and paramagnetic properties of melanin influence its interaction with ionizing radiation

**Figure 1:**
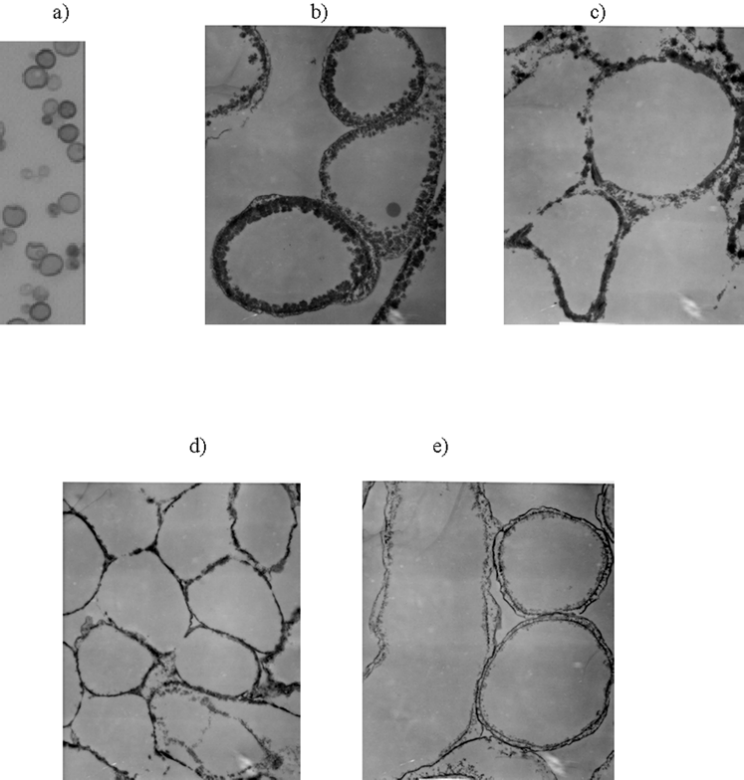
Microscopic images of melanized fungal cells: a) light microscopy image of *C. neoformans* melanin “ghosts”; (b–e) TEM images of *C. sphaerospermum* “ghosts” derived from cells grown on nutrient rich or nutrient-deficient media: b) potato dextrose agar; c) Sabaroud dextrose agar; d) water agar with casein; e) water agar with dextrose. Original magnification: light microscopy image – X 1,000; TEM images – X 13,000.

Our previous work with the human pathogenic fungus *Cryptococcocus neoformans* [24] as well as this study showed that fungal melanin is concentrated in the cell wall and assembled into multiple concentric layers of approximately 100 nm in thickness consisting of closely packed smaller particles [25]. Melanin particles of hollow spherical shape can be isolated from melanized cells by digestion in concentrated acid and have been dubbed “ghosts” because they retain the shape and dimensions of the parent cell. Fig. 1 shows the melanin ghosts of two fungal species investigated in this work - *C. neoformans* which becomes melanized when grown in presence of a melanin pre-cursor such as L-dopa (3,4-dihydroxyphenylalanin) (Fig. 1a) and *Cladosporium sphaerospermum* – an intrinsically melanized fungus found in abundance at the site of nuclear accident in Chernobyl which produces melanin in the variety of growth conditions – from nutrient rich medium to almost complete starvation (Fig. 1b–e).

**Figure 2:**
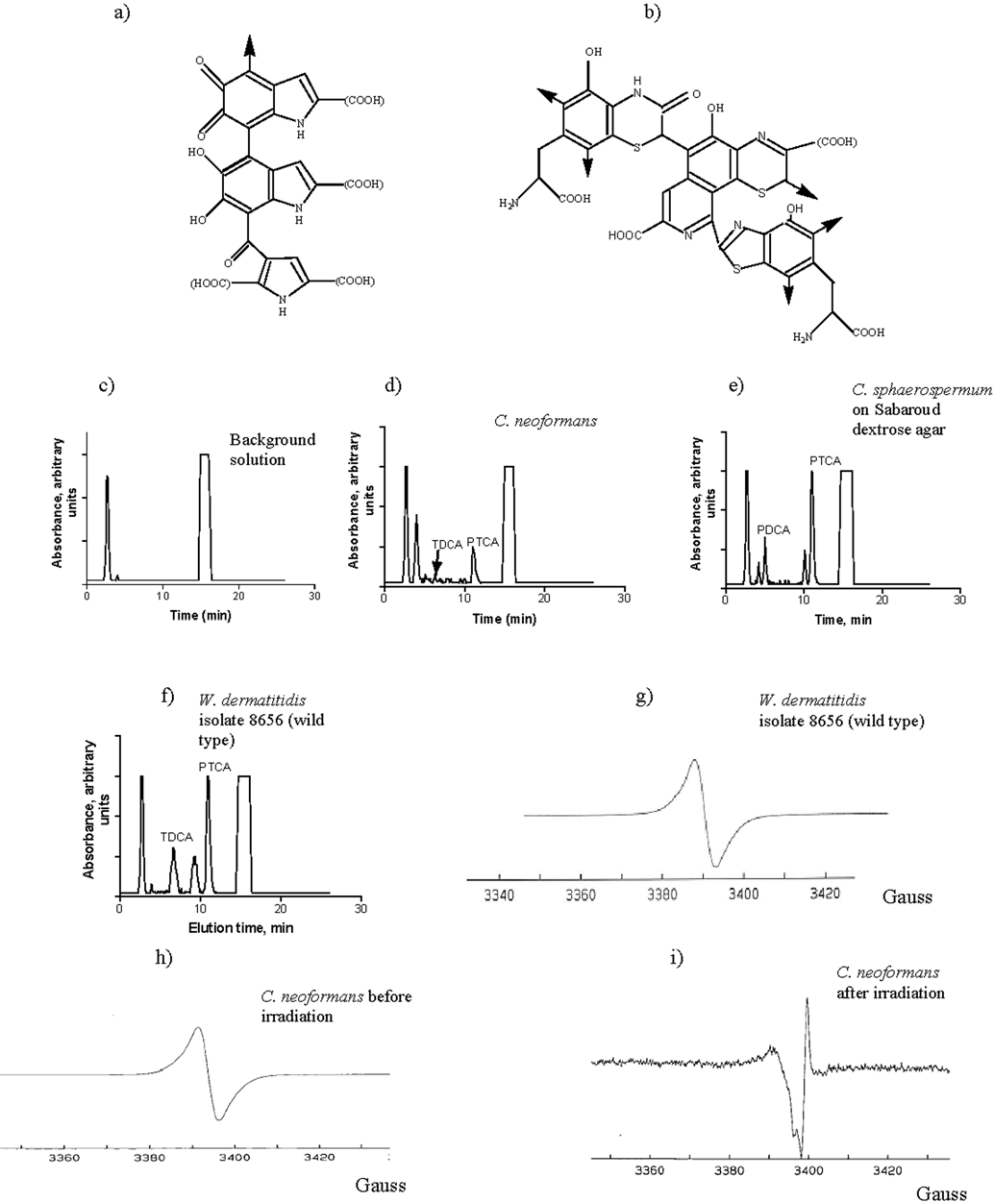
Chemical composition and paramagnetic properties of melanin: a) structure of eumelanin oligomer; b) structure of pheomelanin oligomer; (c – f) HPLC of permanganate-oxidized melanins: c) chromatogram of background solution; d) *C. neoformans;* e) *C. sphaerospermum* grown on Sabaroud dextrose agar; f) *W. dermatitidis* isolate 8656 (wild type); (g–i) ESR spectra: g) *W. dermatitidis* isolate 8656; h) *C. neoformans* before irradiation; i) *C. neoformans* after irradiation with 0.3 kGy dose. PDCA - pyrrole-2,3-dicarboxylic acid; PTCA - pyrrole-2,3,5-tricarboxylic acid; TTCA - 1,3-thiazole-2,4,5-tricarboxylic acid; TDCA - 1,3-thiazole-4,5-dicarboxylic acid. Absorption was monitored at 255 nm and displayed on a linear scale. ESR spectra were obtained by suspending “ghosts” in water except for (h) which was performed in dry state. Ordinate in g–i is the derivative of the ESR absorption in arbitrary units.

**Figure 3:**
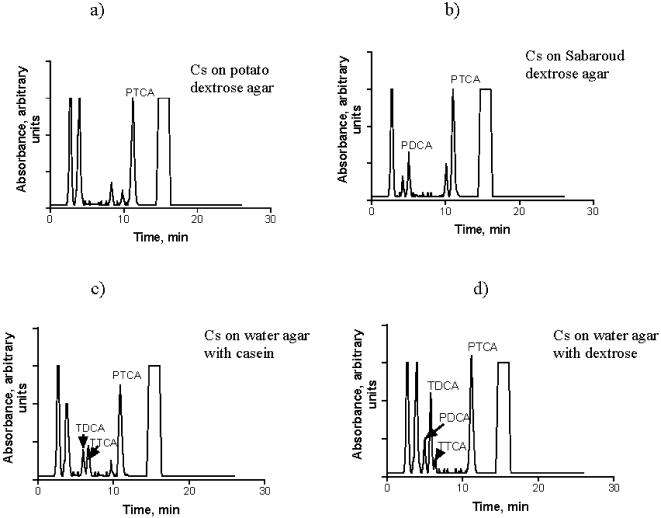
HPLC of melanin derived from *C. sphaerospermum* grown on different substrates: a) potato dextrose agar; b) Sabaroud dextrose agar; c) water agar with casein; d) water agar with dextrose. PDCA - pyrrole-2,3-dicarboxylic acid; PTCA - pyrrole-2,3,5-tricarboxylic acid; TTCA - 1,3-thiazole-2,4,5-tricarboxylic acid; TDCA - 1,3-thiazole-4,5-dicarboxylic acid. Absorption was monitored at 255 nm and displayed on a linear scale. Cs - *C. sphaerospermum*.

In order to investigate the influence of the chemical composition of melanin on its interaction with ionizing radiation, we performed high performance liquid chromatography (HPLC) analysis of melanins from the different fungi used in this study. It must be noted that the structures of both synthetic [17] and natural melanins including fungal melanin are still poorly understood. It is generally accepted, however, that there are two major types of melanin: eumelanin and pheomelanin. Eumelanin is a dark-brown to black pigment composed of 5,6-dihydroxyindole (DHI) and 5,6-dihydroxyindole-2-carboxylic acid (DHICA) monomer units with 6–9% nitrogen and 0–1% sulfur [18], [19] (Fig. 2a). In contrast, pheomelanin is a reddish-brown pigment composed of benzothiazine monomer units with 8–11% nitrogen and 9–12% sulfur [18], [19] (Fig. 2b). When subjected to acidic permanganate oxidation, DHI converts into pyrrole-2,3-dicarboxylic acid (PDCA); DHICA - into pyrrole-2,3,5-tricarboxylic acid (PTCA); and pheomelanin oxidation results in 1,3-thiazole-2,4,5-tricarboxylic acid (TTCA) and 1,3-thiazole-4,5-dicarboxylic acid (TDCA) [18], [19]. We have previously shown that permanganate-oxidized melanin from *C. neoformans* is amenable to HPLC analysis [20], [21]. In this study we performed semi-quantitative assessment of the number of structural subunits of *C. neoformans* melanins. The HPLC of oxidized *C. neoformans* melanin revealed PTCA and TDCA peaks (Fig. 2d) and the presence of these compounds was confirmed by matrix assisted laser desorption/ionization time of flight mass spectrometry (MALDI-TOF). The ratio of PTCA to TDCA was 47.7, which indicates that DHICA subunits predominate in *C. neoformans* melanin. Melanins produced by two different intrinsically melanized fungi *Cladosporium sphaerospermum* (Fig. 2e and Fig. 3) and by *Wangiella dermatitidis* (Fig. 2f) were chemically more diverse than C*. neoformans* melanin, revealing also the peaks assigned to PDCA and peaks at 9–10 min which may be attributed to the small amounts of oxidized 1,8-dihydroxynaphthalene (DHN) melanin.

**Figure 4:**
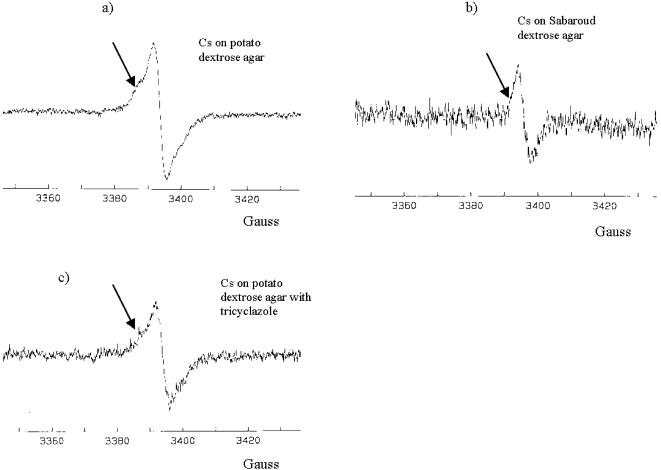
ESR spectra of melanin derived from *C. sphaerospermum* grown on different substrates: a) potato dextrose agar; b) Sabaroud dextrose agar; c) potato dextrose agar impregnated with 25 µg/mL tricyclazole. Differences in *C. sphaerospermum* ESR spectra in comparison with *C. neoformans* are marked with arrows. ESR spectra were obtained by suspending “ghosts” in water. Ordinate is the derivative of the ESR absorption in arbitrary units. Cs - *C. sphaerospermum.*

Electron spin resonance spectroscopy (ESR) of melanized fungi showed the presence a stable free radical population (Fig. 2g–i and Fig. 4) in each of the above fungi, a distinguishing characteristic of melanin [26]. One important indication of melanin interaction with ionizing radiation was a large change in ESR signal of *C. neoformans* dry melanin “ghosts” after they were subjected to 0.3 kGy irradiation and subsequently suspended in water (Fig. 2h and i, respectively).

### Exposure to ionizing radiation and other forms of electromagnetic radiation increases electron transfer properties of melanin

To quantify the effects of ionizing radiation and other forms of electromagnetic radiation on the electron transfer properties of melanin – we irradiated dry *C. neoformans* melanin for 20 and 40 min with 14 Gy/min from a ^137^Cs source and measured its electron transfer properties in the coupled oxidation of NADH and reduction of ferricyanide. In this system, melanin acts as an electron-transfer agent [27], however, the effects of electromagnetic radiation on melanin electron-transfer properties are unknown. Irradiation of melanin for 20 min increased the velocity of the NADH/ferricyanide coupled reaction 3-fold in comparison to that measured for non-irradiated melanin, while 40 min irradiation had an even larger effect, causing a 4-fold increase in velocity (Table 1). When we investigated the influence of other, non-ionizing forms of radiation across the electromagnetic spectrum - heat (infrared radiation), visible light and UV light on the electron-transfer properties of melanin in NADH/ferricyanide coupled reaction – we found that each of these types of radiation increased the ability of melanin to transfer electrons (Table 2). Interestingly, the increase in the electron-transfer properties of melanin was independent of the energy of the incident photons (Table 2).

**Table 1:** NADH-ferricyanide-melanin reaction in presence of untreated and irradiated *C. neoformans* melanin

Sample	Reaction system		
	Ferricyanide+melanin	Ferricyanide+NADH+melanin	
	Ferricyanide reduced	NADH oxidized	Ferricyanide reduced
Untreated melanin	40 nmol	37 nmol	75 nmol
	V^1^ = 9 nmol/min		V = 30 nmol/min
Irradiated melanin, 20 min	60 nmol	100 nmol	200 nmol
	V = 13 nmol/min		V = 80 nmol/min
Irradiated melanin, 40 min	170 nmol	150 nmol	300 nmol
	V = 38 nmol/min		V = 120 nmol/min

1 V - initial velocity is expressed in nanomoles of ferricyanide reduced per min.

**Table 2:** Increase in electron-transfer properties of melanin in NADH/ferricyanide coupled reaction after exposure to different forms of electromagnetic radiation

Radiation type	Photon energy, eV	Increase in initial velocity in NADH/ferricyanide reaction
Ionizing radiation from 137-Cs source	661,000	4.0
UV, 254 nm	4.7	3.9
Visible light, 250 W	3	4.0
Heat, 75°C	0.1	3.7
photosynthesis	3	N/A

50 µg of *C. neoformans* melanin was used in the reactions; melanin was subjected to 40 min treatment, placed into dry ice following treatments and taken up in the ferricyanide solution immediately before measurements. To exclude contribution of heat component during irradiation of melanin with 250 W light, the samples were placed in 25°C water bath.

### Metabolic activity of melanized and non-melanized cells in the presence of electromagnetic radiation

**Figure 5:**
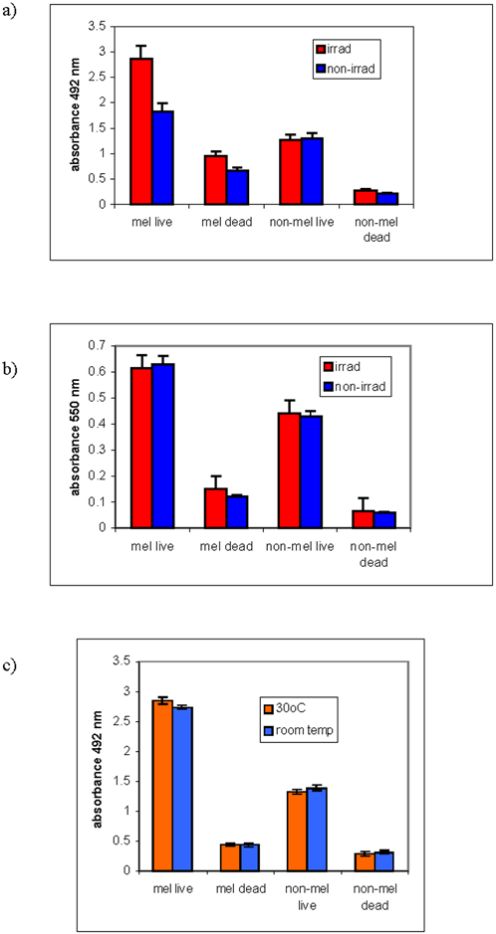
The influence of ionizing radiation or heat on the metabolic activity of melanized and non-melanized *C. neoformans* cells. a, b) irradiated and non-irradiated cells: a) XTT; b) MTT. c) XTT of cells grown at room temperature (22°C) or at 30°C. The cells were kept in the dark while being exposed to ionizing radiation or different temperatures. Following the exposure, XTT or MTT reagents were added to the samples and absorbance was measured at 492 or 550 nm for XTT and MTT, respectively.

We investigated whether the changes in electron transfer properties of melanin post exposure to ionizing radiation (high-energy photons, see Table 2) may also be observed in melanized cells exposed to ionizing radiation. The metabolic activity of *C. neoformans* cells was evaluated with 2,3-bis(2-methoxy-4-nitro-5-sulfophenyl)-5-[(phenylamino) carbonyl]-2H-tetrazolium hydroxide (XTT assay) [28] and 2-(4,5-dimethyl-2-thiazolyl)-3,5-diphenyl-2H-tetrazolium bromide (MTT assay). The use of XTT and MTT assays in parallel can help to define the location of the melanin-mediated electron transfer in the cells since positively charged MTT is taken into the cells via the plasma membrane potential and is reduced intracellularly; while the negatively charged XTT is largely cell-impermeable and its reduction occurs extracellularly, at the cell surface [29]. The melanized and non-melanized *C. neoformans* cells were exposed to ionizing radiation in the dark at 22°C overnight. The irradiation was performed in a constant field of 0.05 mGy/hr, a non-fungicidal radiation dose that is comparable to the doses inside the Chernobyl reactor [6]. Following exposure to radiation, the XTT or MTT reagents were added to the samples and absorbance was measured at 492 or 550 nm, respectively. The XTT assay showed significant increase in electron-transfer events in the irradiated melanized cells in comparison with non-irradiated melanized or irradiated non-melanized cells (Fig. 5a). Increased absorbance at 492 nm was also observed for dead (heat killed) melanized cells in comparison to non-melanized ones, showing that melanin can reduce XTT reagent by itself (Fig 5a). Irradiation of dead cells caused significant increase in the XTT reduction, thus confirming our hypothesis that radiation enhances electron-transfer properties of melanin. In contrast, there was no difference between the irradiated and non-irradiated melanized and non-melanized cells subjected to MTT assay (Fig. 5b). The difference between the MTT and XTT assays may be explained by the occurrence of radiation-related melanin-mediated electron transfer events near cell wall where melanin is located that led to higher XTT reduction in irradiated melanized samples. Interestingly, both irradiated and non-irradiated melanized cells showed higher activity by MTT assay than non-melanized cells (Fig. 5a, b). Given that melanization is associated with reduced pore size that could reduce passive nutrient uptake [25] and that melanin is synthesized from highly reactive cytotoxic intermediates of the oxidation of L-Dopa - it is possible that melanization requires a higher metabolism for cell survival.

In another series of experiments, the melanized and non-melanized cells were grown overnight in the dark at room temperature (22°C) or 30°C. Melanized cells demonstrated increased XTT reduction activity at both temperatures in comparison with non-melanized controls (Fig. 5c), and increasing the temperature to 30°C caused a statistically significant increase in XTT reduction in melanized cells (P<0.05) while a small decrease was observed for non-melanized cells. Overall, these experiments showed the increase in electron transfer properties of melanin in melanized cells post exposure to ionizing radiation and to less extent - to heat.

### Ionizing radiation enhances growth and ^14^C-acetate uptake of melanized *C. neoformans* cells

**Figure 6:**
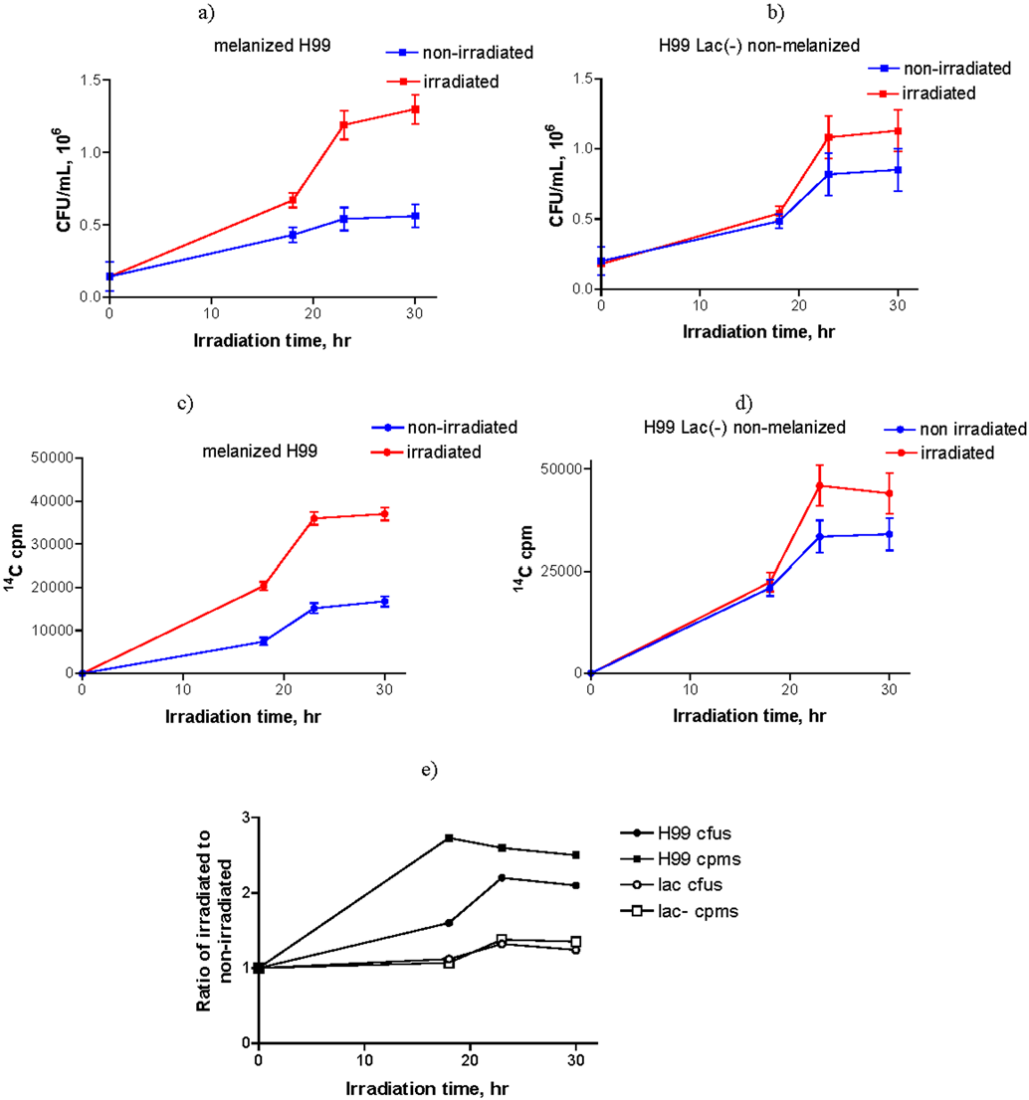
Growth and incorporation of ^14^C-acetate by melanized C. neoformans H99 cells and non-melanized Lac(-) H99 cells lacking the laccase enzyme under conditions of limited nutrients supply in a radiation field of 0.05 mGy/hr or at background radiation level. a) growth of melanized H99 cells; b) growth of non-melanized Lac(-) H99 cells; c) incorporation of ^14^C-acetate into melanized H99 cells; d) incorporation of ^14^C-acetate into non-melanized Lac(-) H99 cells; e) ratio of irradiated to non-irradiated cells CFUs and cpms ratios (normalized CFUs and cpms) for melanized H99 and non-melanized Lac(-) H99 cells.

To expand the observations of the influence of irradiated melanin on the growth of melanized cells, we measured the growth of melanized and non-melanized *C. neoformans* cells supplied with limited nutrients and placed into the radiation flux. To maintain a steady population of melanized cells, we used the same H99 strain of *C. neoformans* as in XTT/MTT experiments since it is capable of making melanin when maintained with L-Dopa while its laccase disrupted [Lac(-)] mutant is incapable of melanization [30]. The cells were grown into stationary phase up to 30 hr. There were significantly more (P = 0.006) CFUs for irradiated melanized wild type H99 samples at 18, 23 and 30 hr than for non-irradiated samples (Fig. 6a), while the difference in CFUs at 18 hr between irradiated and non-irradiated Lac(-) mutant was not significant (Fig. 6b). Lac(-) without radiation in the presence of L-dopa grows better than wild type H99 (Fig. 6a and b). There was also a slight increase in the CFU's of irradiated Lac(-) cells at 23 and 30 hr. However, the crucial difference between the wild type H99 and Lac(-) cells is that the exposure to ionizing radiation produced approximately 2.5 times more CFUs in irradiated melanized cells than in non-irradiated melanized controls, while irradiation of Lac(-) cells resulted only in a 1.1-fold increase in CFUs (Fig. 6e). The dry weight measurements performed at 20 hr showed a consistent and significant 6.5% increase for irradiated melanized samples (P = 0.02) while there was no difference in weight for the mutant strain after irradiation. The relatively small yet significant increase in dry weight of the melanized cells is a result of the high percentage of immature cells, with smaller capsules synthesized *de novo* in the dividing melanized irradiated cell culture. In this regard, a cell diameter that is one-half to one-third of that for a mature cell results in a 8- and 26-fold decrease in cell mass, respectively. Quantification of whole cell sizes using India ink stained cells showed that proximately 50% of melanized irradiated cells had volumes 2 times smaller than those in the irradiated Lac(-) mutant population (results not shown), accounting for the relatively small increase in the dry weight of the melanized H99 samples in comparison to their larger increase in CFUs.

To obtain additional evidence that exposure to ionizing radiation enhanced melanized cell growth, we measured the incorporation of a ^14^C-labeled carbon source (acetate) into melanized and non-melanized *C. neoformans* cells with and without radiation flux. In the photosynthesis field the incorporation of ^14^C-acetate in bacteria subjected to visible light is considered to be indicative of their photoheterotrophic capabilities [31]. We measured a lower absolute uptake of ^14^C-acetate by wild type H99 compared to Lac(-) cells (Fig. 6c,d). There was no incorporation of ^14^C-acetate into heat killed melanized or non-melanized cells, which excludes the possibility that radiation promoted the passive absorption of ^14^C-acetate on melanin. Importantly, when melanized and non-melanized Lac(-) H99 cells were incubated with ^14^C-acetate with and without radiation – there was almost 3 times more incorporation of ^14^C-acetate into irradiated melanized cells than into non-irradiated melanized cells, while the ratio of ^14^C-acetate incorporation into irradiated to non-irradiated Lac(-) cells was only slightly higher than 1 (Fig. 6c,d and e). Overall, these results demonstrate that the presence of melanin contributes to the enhancement of cellular growth upon exposure to ionizing radiation in conditions of limited nutrients.

### Intrinsically melanized fungi C. *sphaerospermum* and *W. dermatitidis* manifested enhanced growth in radiation flux

**Figure 7:**
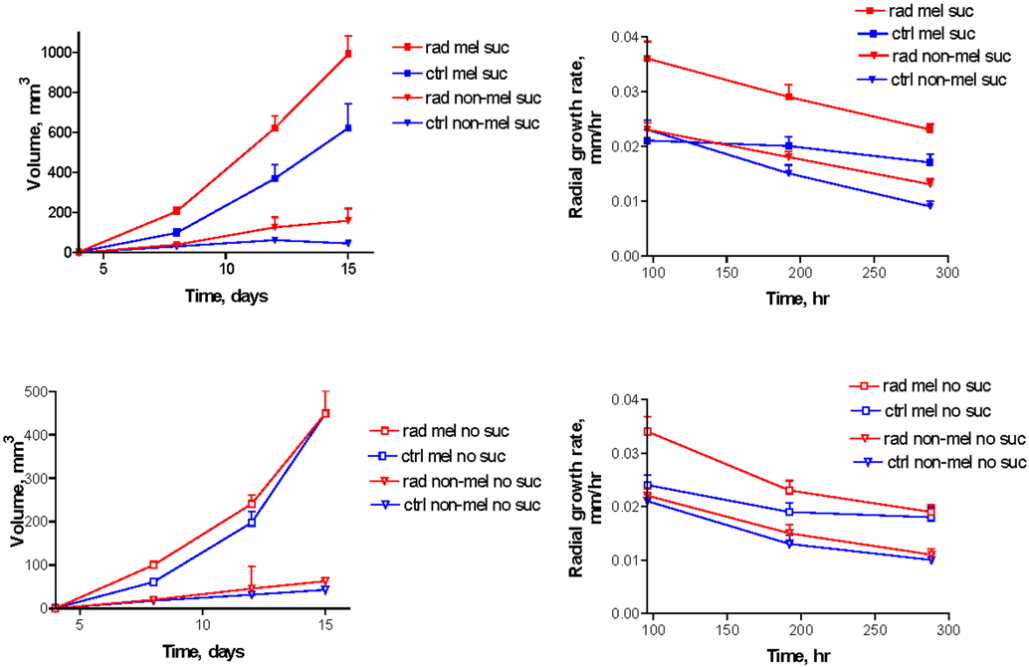
Survival of non-melanized and melanized C. sphaerospermum cells following exposure to external gamma rays: average volume (left side plates) and radial growth rate (right side plates) of melanized and melanin-deficient *C. sphaerospermum* colonies grown on agar plates with (top panels) or without (bottom panels) sucrose in a radiation field of 0.05 mGy/hr or at background radiation level (control). rad - irradiated, ctrl - control, mel - melanized, suc- sucrose added. The volume of half-sphere was calculated as V/2 = π/12 d^3^. Radial linear growth rate of *C. sphaerospermum* colonies was calculated as K = (R_t_−R_o_)/(t−t_o_), where K is radial linear growth rate, mm/hr; R_t_ and R_o_ - colony radii at time t and time t_o_, respectively.

To accrue additional data that ionizing radiation can promote enhanced growth of melanized fungi we extended our observations to two intrinsically melanized fungal species. In contrast to *C. neoformans* which must be supplied with laccase substrate like L-Dopa for melanization - both C. *sphaerospermum* and *W. dermatitidis* synthesize melanin without the need for exogenous precursors. Initially we selected *C. sphaerospermum* because this fungus is a dominant species inhabiting the damaged reactor at Chernobyl [6]. *C. sphaerospermum* was placed in a constant radiation field of 0.05 mGy/hr and colony diameters were measured for 15 days. We supplemented water agar with minimal media containing sources of carbon and mineral salts, and, in one condition, - with a limited amount of sucrose (Fig.7). We observed melanin production by this fungus even when the amounts of nutrients and energy sources in the media were very low or absent (Fig.1b–e). To evaluate the contribution of melanin to the enhanced growth in the radiation flux - we generated non-melanized cells by growing the fungus in the presence of tricyclazole, a specific inhibitor of pentaketide synthetic pathway of 1,8-dihydroxynaphthalene (DHN) melanin [22]. Exposure of melanized cells to radiation was associated with increased growth of colonies in all conditions. On agar with sucrose, the irradiated melanized colonies of *C. sphaerospermum* grew significantly more in regard to their volume and faster as shown by their radial growth rate than control melanized or control melanin-deficient ones (Fig. 7). Irradiated melanized colonies grew more than irradiated melanin-deficient cells demonstrating that radiation is not inducing fungal agarase - an enzyme that breaks down agar into possible nutrients, or simply altering the agar to provide nutrients. The same trend was observed for cells grown without sucrose - the largest and fastest growing colonies were observed for irradiated melanized cells in comparison with the other 3 controls. All colonies grown without sucrose were smaller than those grown with this nutrient. The enhanced growth as a consequence of better survival of melanized fungi in radiation field in comparison with non-melanized ones can be also ruled out by inherent radioresistance of fungi, as the doses delivered to *C. sphaerospermum* were less than 0.0001% of the fungicidal dose [8], [9]. The conclusion from these experiments is that melanin was produced by fungi even when the amounts of nutrients and energy sources in the media were very low of absent; and the irradiated melanized cells experienced increased growth even in the conditions of starvation.

The *C. sphaerospermum* system had two limitations: 1) generation of non-melanized cells required tricyclazole, a compound that could arguably affect other metabolic processes; and 2) this fungus is a mold and its hyphal cells aggregated and this precluded measuring growth by standard CFUs. Although the results showing larger colony radial growth were strongly suggestive of increased fungal mass, larger colonies could conceivably have resulted from differences in cellular packing or swelling. Consequently, we used a third organism to study the effect of irradiation on melanized fungal growth. *Wangiella dermatitidis*, an intrinsically melanized human pathogenic fungus that exists predominantly as a yeast form in vivo and at 37°C, was selected for further study since an albino strain of *W. dermatitidis* (*wdpks1Δ-1*; lacking the polyketide synthase gene *WdPKS1* responsible for generating melanin) and its complemented strain (*wdpks1*Δ*-1*-501) recently became available [23]. This fungus, which also produces fewer multicellular forms in vitro in comparison with *C. sphaerospermum*, allowed us to quantify the effect of radiation on cell growth by CFUs instead of measuring colony diameters.

**Figure 8:**
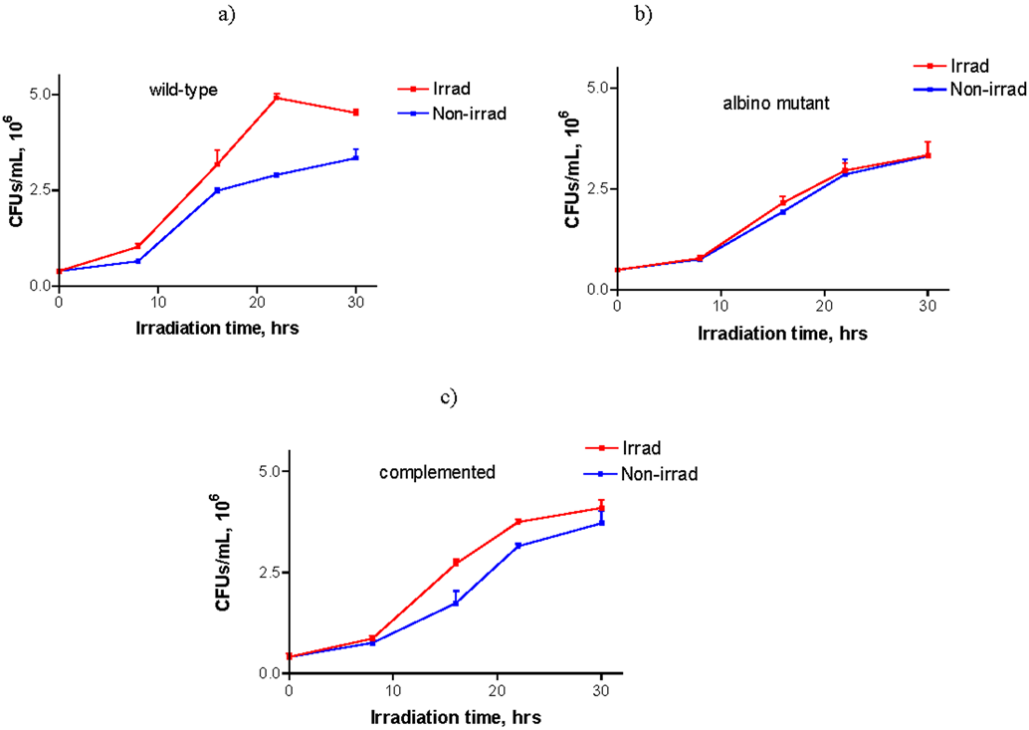
Growth of *W. dermatitidis*. a) wild type 8656; b) albino mutant *wdpks1Δ-1* with a disrupted polyketide synthase gene; c) a strain complemented with wild type gene *wdpks1*. The cells were grown under conditions of limited nutrients in a radiation field of 0.05 mGy/hr or at background radiation level. The cells were exposed to radiation for various times and plated for CFUs on YPD.

Exposure of *W. dermatitidis* cells to ionizing radiation resulted in significantly more cells being produced, as measured by CFUs, for the melanized strains (P<0.01) than for the non-irradiated melanized control cells or the irradiated *wdpks1Δ-1* albino mutant strain (Fig. 8a–c). At 16, 22 and 30 hrs of irradiation the two melanin-containing strains of *W. dermatitides* had more colonies than the amelanotic mutant strain. As in the case of *C. neoformans,* some increased growth was observed in the irradiated albino mutant in comparison with non-irradiated albino cells at 16 hrs (Fig. 8b). Wild type and complemented cells exposed to radiation manifested significantly shorter doubling times (Table 3) in comparison with non-irradiated controls while there was no statistical difference for the albino mutant. Dry weight experiments conducted for all three *W. dermatitides* strains irradiated for 20 hr showed 8–10% (P = 0.02) increase in dry weight for melanized wild type and complemented strains in comparison with non-irradiated controls. As for *C. neoformans* - the lower percentage increase in dry weight in comparison with the percentage increase in CFUs (40 and 20% for 20 hr time point for wild type and complemented strains, respectively) can be explained by the fact that 50% of the cells in the melanized cultures were newly formed with volumes 2 times lower (results not shown) than that of the irradiated more mature albino cells.

**Table 3:** Doubling times for *W. dermatitidis* grown in nutrient-deficient medium with and without radiation. The means and SEM of 4 experiments are presented.

Strain	Doubling time, hr		
	irradiated	control	P value*
wild type	6.5 (0.1)	7.4 (0.2)	0.02
mutant	9.8 (1.0)	10.8 (1.8)	0.7
complemented	6.8 (0.1)	7.3 (0.1)	0.01

* Wilcoxon non-parametric test for unpaired data was performed for each strain to compare doubling times for irradiated and control samples. Doubling time = ln 2/((ln(A/A_o_)/t), where A-amount of cells at time t, A_o_ – amount of cells at time 0.

## Discussion

To investigate the influence of ionizing radiation on the electron-transfer properties of melanin and on the growth of melanized fungi, we performed multiple physico-chemical tests and in vivo experiments with 3 genetically diverse fungi. HPLC results which reveal the chemical structure of melanin from different fungi are important for understanding the electronic properties of melanin. The number of electrons per gram is an important contributor to the attenuation properties of a material at the energy levels where the Compton effect predominates [32]. Compton scattering predominates for chemical elements with low atomic numbers such as C, N, O and S [32], which constitute melanin. In Compton scattering, transfer of a photon energy to matter occurs via a cascade of interactions, where the energy of the incident photon is transferred to high-energy electrons, and to secondary photons of progressively lower energy until the photoelectric effect takes place. Thus, the existence of structures composed of electron-rich covalently linked aromatic motifs could explain radiation scattering properties of melanins. Furthermore, the higher number of electrons in oligomers of pheomelanin relative to eumelanin – 388 versus 287 – could result in better scattering properties of pheomelanin.

The high-energy electrons generated by Compton scattering are ultimately responsible for the radiobiologic effects caused by gamma radiation by either direct interaction with DNA or through radiolysis of water in the cells, a process that results in the formation of reactive short-lived free radicals capable of damaging DNA. Stable free radicals in melanin may interact with these high-energy electrons and prevent them from entering a cell, thus enabling melanin to function as a radioprotector. The Compton electrons may then undergo secondary interactions with melanin molecules with their energy gradually lowered by melanin.

When performing experiments on measuring the growth and incorporation of ^14^C-acetate into irradiated and non-irradiated melanized *C. neoformans* cells and its non-melanized Lac(-) mutant, we noted that non-irradiated Lac(-) cells grew better and incorporated more ^14^C-acetate than non-irradiated melanized cells (Fig. 6b,d). Although the basis for this difference is not understood it may be related to melanin limited porosity in the wild type melanized strain, since pore size decreases with culture age [25] and might reduce the availability of nutrients. Also, the process of melanization involves an oxidation reaction with generation of toxic intermediates which may impose a certain metabolic cost that could translate into slower in growth relative to Lac(-) cells. The slight increase in the CFU's and ^14^C-acetate incorporation of irradiated Lac(-) cells at 23 and 30 hr is probably due to the well documented phenomenon that very low doses of ionizing radiation can stimulate cell proliferation [33], [34]. However, the crucial difference between the wild type H99 and Lac(-) cells is that the exposure to ionizing radiation resulted in 2.5 times more CFUs and almost 3 times more incorporation of ^14^C-acetate in irradiated melanized cells than in non-irradiated melanized controls, while irradiation of Lac(-cells resulted only in a 1.1-fold increase in CFUs and ^14^C-acetate incorporation (Fig. 6e).

Melanins are unique biopolymers that protect living organisms against UV and ionizing radiation and extreme temperatures. The electronic complexity of melanins allows them to scatter/trap photons and electrons, which was evidenced in this study by the following observations: 1) changes the electronic structure of melanin post radiation exposure as measured by amplitude changes in the ESR signal; 2) electron transfer properties of melanin in the NADH oxidation/reduction reaction increased 4-fold after melanin irradiation. The ability of radiation to preferentially enhance the growth of melanized fungi is implied by the following observations made in this study: melanized *C. neoformans* and *W. dermatitidis* cells exposed to levels of radiation approximately 500 times higher than background grew significantly faster as indicated by the presence of more CFUs, greater biomass as shown by dry weight measurements and/or relative incorporation of more ^14^C-acetate than non-irradiated melanized cells. Furthermore, comparative analysis of MTT/XTT reduction assays revealed that radiation-induced effects on the electron transfer properties of melanin were localized to the extracellular space thus establishing a spatial relationship between the site for electron-transfer events and the location of the melanin pigment. In addition, we recorded radiation-induced effects on the growth of melanized *C. sphaerospermum* cells under limited nutrients conditions. Hence, we observed that radiation increased the growth of melanized cells relative to non-melanized cells using three fungal species and four measures of cell growth.

The literature already contains some indirect evidence for the notion that radiation can enhance the growth of melanized microorganisms. For example, the melanotic fungus *C. cladosporioides* manifests radiotropism by growing in the direction of radioactive particles and this organism has become widely distributed in the areas surrounding Chernobyl since the nuclear accident in 1986 [7]. Both in the laboratory and in the field several other species of melanized fungi grew towards soil particles contaminated with different radionuclides, gradually engulfing and destroying those particles [35], [36]. In addition, there are recent reports that certain life forms can utilize non-conventional forms of energy - microbes in geothermal vents at the bottom of the ocean can harvest thermal radiation as an energy source [37] while some microorganisms living in mines exploit energy from radiolysis of water [38]. On the basis of these precedents and the results of this study we cautiously suggest that the ability of melanin to capture electromagnetic radiation combined with its remarkable oxidation-reduction properties may confer upon melanotic organisms the ability to harness radiation for metabolic energy. The enhanced growth of melanotic fungi in conditions of radiation fluxes suggests the need for additional investigation to ascertain the mechanism for this effect.

## Methods

### Microorganisms

American Type Culture Collection (ATCC, Rockville, MD) strain *C. neoformans* H99 and its laccase lacking mutant Lac(-) (kind gift from Dr. A. Idnurm, Duke University, NC) were used in all experiments. *C. neoformans* was grown in Sabouraud dextrose broth (Difco laboratories, Detroit, MI) for 24 hrs at 30°C with constant shaking at 150 rpm. Melanized *C. neoformans* cells were generated by growing the fungus in minimal medium with 1 mM 3,4-dihydroxyphenylalanine (L-dopa) for 5–10 days. *C. sphaerospermum* (ATCC, VA), an intrinsically melanized fungus, was grown on potato dextrose agar (Becton, Dickinson and Company) for 15 days. Approximately 1,000 *C. sphaerospermum* cells were plated on different media: water agar impregnated with minimal media (no glucose) and casein; agar dissolved in minimal media (no glucose) and 40 g/L dextrose; potato dextrose agar (Becton, Dickinson and Company); potato dextrose agar impregnated with 25 ug/mL tricyclazole; and Sabaroud dextrose agar. The laboratory wild-type strain of intrinsically melanized *W. dermatitidis* 8656 {ATCC 34100 [*Exophiala dermatitidis* CBS 525.76]} a strain with a disrupted polyketide synthase gene *wdpks1Δ-1*, and its complemented isolate (*wdpks1*Δ*-1*-501) were a kind gift from Dr. P. Szaniszlo (The University of Texas at Austin, Austin, TX). Routine propagation of these strains was in YPD [2% peptone, 1% Bacto Yeast extract, and 2% dextrose] at 37°C with shaking at 150 rpm.

### Isolation and purification of fungal melanins

Fungal cells were suspended in 1.0 M sorbitol-0.1 M sodium citrate (pH 5.5). Lysing enzymes from *Trichoderma harzarium* (Sigma Chemical Co.) were added to the suspension at 10 mg/mL and the suspensions were incubated overnight at 30°C. Protoplasts were collected by centrifugation, and incubated in 4.0 M guanidine thiocyanate overnight at room temperature. The resulting particulate material was collected by centrifugation, and Proteinase K (1.0 mg/mL) in reaction buffer (10.0 mM tris, 1.0 mM CaCl_2_, 0.5% SDS) was added to the particles followed by overnight incubation at 37°C. The particles were boiled in 6.0 M HCl for 1 hour. Finally, the resulting material (“ghosts”) was washed with PBS, dialyzed against deionized water for 2 days and dried in air at 65°C overnight.

### Transmission electron microscopy (TEM)

Samples were processed at the Analytical Imaging Facility, AECOM. The *C. neoformans, C. sphaerospermum* and *W. dermatitidis* “ghosts” or cells were frozen under high pressure using a Leica EMpact High Pressure Freezer (Leica Microsystems, Austria). Frozen samples were transferred to a Leica EM AFS Freeze Substitution Unit and freeze substituted in 1% osmium tetroxide in acetone. They were brought from −90°C to room temperature over 2–3 days, rinsed in acetone and embedded in Spurrs epoxy resin (Polysciences,Warrington, PA.). Ultrathin sections of 70–80 nm were cut on a Reichert Ultracut UCT, stained with uranyl acetate followed by lead citrate and viewed on a JEOL (Tokyo, Japan) 1200EX transmission electron microscope at 80 kV.

### Oxidation of melanins and HPLC of oxidized melanins

The melanin “ghosts” were subjected to acidic permanganate oxidation as described in [18]–[21]. The pyrrole-2,3,5-tricarboxylic acid (PTCA), pyrrole-2,3-dicarboxylic acid (PDCA), 1,3-thiazole-2,4,5-tricarboxylic acid (TTCA) and 1,3-thiazole-4,5-dicarboxylic acid (TDCA) used as standards were a kind gift from Dr. K. Wakamatsu of Fujita Health University of the Health Sciences, Toyoake, Japan. The oxidation products were analyzed by HPLC using a Shimadzu LC-600 liquid chromatograph, Hamilton PRP-1 C_18_ column (250×4.1 mm dimensions, 7 µm particle size), and Shimadzu SPD-6AV UV detector. The mobile phase was 0.1% trifluoroacetic acid in water (solvent A) and 0.1% trifluoroacetic acid in acetonitrile (solvent B). At 1.0 mL/min, the elution gradient was (min, %B): 0, 0; 1, 0; 12, 25; 14, 25; 16, 0. The UV detector was set at a 255 nm absorbance.

### MALDI mass spectrometry

The major peaks generated during chromatography of oxidized melanins were collected and analyzed by MALDI-TOF mass spectrometry in positive pressure mode on PE-Biosystems Mariner mass spectrometer. A peptide mixture with molecular weights of 1059.56, 1296.68 and 1672.95 in 2,5-dihydroxybenzoic acid matrix was used for calibration.

### Electron spin resonance spectroscopy (ESR)

The ESR of melanin “ghosts” was performed on ER 200D EPR/ENDOR spectrometer with ESP 300 upgrade (Brucker Instruments, Inc. Billerica, MA). ESR spectra were obtained by suspending “ghosts” in water. ESR spectra of *C. neoformans* “ghosts” were also obtained in dry state before irradiation with 0.3 kGy and the “ghosts” were subsequently suspended in water and ESR was repeated.

### NADH-ferricyanide reaction in the presence of untreated and irradiated *C. neoformans* melanin

The ability of melanin to oxidize or reduce NADH and ferricyanide was determined spectrophotometrically as in [27]. The absorbance of NADH was monitored at 340 nm, of ferricyanide - at 420 nm. Fifty µg of *C. neoformans* melanin was used in the reactions; dry melanin was subjected to 20 and 40 min irradiation with the 137-Cs source at a dose rate of 14 Gy/min; put into dry ice following irradiation and taken up in the ferricyanide solution immediately before measurements.

### Determination of metabolic activity of melanized and non-melanized *C. neoformans* cells subjected to ionizing radiation or different temperatures by XTT and MTT assays

Melanized and non-melanized *C. neoformans* cells were washed, suspended in PBS and their concentration was adjusted to 10^8^ per mL. To account for the possibility of melanin itself changing the reaction through electron transfer or solubility/retention of formazan product [28] - the aliquots of both melanized and non-melanized cells were heat-killed at 65°C (water bath) for one hour and used as controls. 10^7^ cells were placed into the wells in 96 well plates, 5 wells per each condition. The plates were covered with foil to exclude any light effects and incubated overnight at room temperature (22°C), at 30°C, or at 22°C in a constant radiation field of 0.05 mGy/hr. For XTT (2,3-bis(2-methoxy-4-nitro-5-sulfophenyl)-5-[(phenylamino) carbonyl]-2H-tetrazolium hydroxide) assay 54 µL (XTT)/menadinone was added to each well, the plates were covered with foil, shaken for 2 minutes, and incubated at 37°C for 2 hrs. The absorbance was read at 492 nm (Labsystem Multiskan, Franklin, MA). For MTT (2-(4,5-dimethyl-2-thiazolyl)-3,5-diphenyl-2H-tetrazolium bromide) assay, the MTT solution in PBS was added to the wells with the cells, so that the final MTT concentration became 0.5 mg/mL. After incubation at 37°C, the contents of the wells was spun down at 2,000 rpm, supernatant was discarded, followed by addition of 200 µl 0.04 M HCl in absolute isopropanol to each sample. The samples were transferred into the 96-well plate and the absorbance was read at 550 nm.

### Exposure of *C. neoformans* to ionizing radiation under limited nutrient conditions, ^14^C-acetate incorporation and dry weight measurements

H99 wild type and Lac(-) mutant cells were grown as above. Melanization of H99 was achieved by incubation in 1mM L-Dopa/minimal medium (1/200) in the dark at 30°C, at 150 rpm. The cells were washed with essential salts solution (3 g/L NaNO_3_, 1 g/L K_2_HPO_4_, 1 g/L MgSO_4_.7H_2_O, 0.5 g/L KCl, 0.003 g/L thiamine, 5.3 g/L NH_4_Cl), pelleted and taken up in 1 mM Na acetate solution in essential salts spiked with 0.1 µCi/mL ^14^C-acetate. The cell concentration was adjusted to 10^5^ cells/ml, 1 mL samples of each strain were placed in 1.5 mL Eppendorf tubes (4 samples per time point) and subjected either to the background level of radiation or to a radiation field of 0.05 mGy/hr created by ^188^Re/^188^W isotope generator for up to 30 hr at 30°C. The cell uptake of ^14^C-acetate was quantified by counting the tubes in a scintillation counter, spinning cells, separating supernatant and counting the cell pellet again. The cells were also plated for CFUs. For dry weight experiments 5 mL of cells at 4×10^7^ cells/mL cell density were irradiated for 20 hr at 30°C, filtered through pre-weighed 0.2 µ filters, the filters were dried and weighed again.

### Exposure of *C. sphaerospermum* to ionizing radiation under limited nutrients conditions

Melanized and non-melanized (tricyclazole-treated) *C. sphaerospermum* were grown on BBL Sabouraud dextrose agar for 15 days, harvested and plated on water agar prepared by mixing agar powder with minimal media lacking glucose. Four-sectional, 100×15 mm dishes were used. Identical plates were made for irradiated and control samples. Five mL of the above agar was added to each of the left two sections of each plate. These sections were called “no sucrose” sections. Five mL volumes of sucrose-containing agar (prepared by mixing agar powder with 100 mg/L sucrose as described in [6] and with minimal media lacking glucose) was added to the right two sections of each plate. Non-melanized *C. sphaerospermum* was added to the top compartments and melanized *C. sphaerospermum* was added to the bottom compartments of each plate. Agar in the top compartments where non-melanized *C. sphaerospermum* was plated contained 25 µg/mL tricyclazole to inhibit melanization. Approximately 20 cells per section were plated. All plates were prepared in duplicate. The plates were wrapped in foil and exposed continuously to 0.05 mGy/hr created by ^188^Re/^188^W isotope generator for 15 days while control plates were exposed only to background radiation (10^−4^ mGy/hr). The colonies were counted and measured daily.

### Exposure of *W. dermatitidis* to ionizing radiation under limited nutrients conditions and dry weight measurements

Before radiation exposure, wild type, albino mutant and complemented strains of *W. dermatitidis* were cultured in the following manner using the modified procedure from [39]: frozen cells were diluted and cultured in 20 mL YPD at 37°C with shaking at 150 rpm for 48 hrs, then diluted in YPD to 10^6^ cells/mL and grown for another 48 hrs. At the end of the 2^nd^ 48 hr period both wild type and complemented strains developed dark coloration while albino mutant cells were light yellow. The cells were again diluted to 10^6^ cells/mL and cultured for 24 hrs, and this procedure was repeated once. Next, the cells were again diluted and grown for 24 hours in minimal chemical media (3 g/L NaNO_3_, 1 g/L K_2_HPO_4_, 1 g/L MgSO_4_.7H_2_O, 0.5 g/L KCl, 0.003 g/L thiamine, 5.3 g/L NH_4_Cl, pH of 6.5) supplemented with 120 mg/L sucrose as a carbon source. The wild type and complemented strains maintained their dark color and the albino mutant remained light yellow. The cells were collected, washed, and diluted to 5×10^5^ cells/mL in the minimal medium. One ml aliquots in 1.5 ml microfuge tubes were placed at 37°C in the dark without shaking, either in the cell incubator with the background level of radiation or in a radiation field of 0.05 mGy/hr created by ^188^Re/^188^W isotope generator. For each time point, triplicate samples were used. After 16, 22 and 30 hrs of exposure the cells were plated on YPD agar for 4 days at room temperature for determination of CFUs. The cells entered stationary phase around 30 hrs. For dry weight determinations, 20 mL of each strain in essential salts solution supplemented with 120 mg/mL sucrose at cell density of 10^7^ cell/mL were irradiated for 20 hrs or kept at background radiation level then filtered through pre-weighed 0.2 µ filters that were dried and weighed again.

### Statistical analysis

Wilcoxon non-parametric test for unpaired data was performed to analyze the differences in CFUs and ^14^C-acetate uptake. Differences were considered statistically significant when P values were<0.05.
